# Light sheet microscopy reveals more gradual light attenuation in light-green versus dark-green soybean leaves

**DOI:** 10.1093/jxb/erw246

**Published:** 2016-06-20

**Authors:** Rebecca A. Slattery, Aleel K. Grennan, Mayandi Sivaguru, Rosangela Sozzani, Donald R. Ort

**Affiliations:** ^1^Department of Plant Biology, University of Illinois at Urbana-Champaign, 1206 West Gregory Drive, Urbana, IL 61801, USA; ^2^Carl R. Woese Institute for Genomic Biology, University of Illinois at Urbana-Champaign, 1206 West Gregory Drive, Urbana, IL 61801, USA; ^3^Department of Plant and Microbial Biology, North Carolina State University, 2115 Gardner Hall, Box 7612, Raleigh, NC 27695, USA; ^4^Global Change and Photosynthesis Research Unit, United States Department of Agriculture, 1206 West Gregory Drive, Urbana, IL 61801, USA

**Keywords:** Chlorophyll, *Glycine max*, leaf light environment, light sheet microscopy, light use efficiency, photosynthesis, photosynthetic efficiency, soybean.

## Abstract

Light wavelengths preferentially absorbed by chlorophyll (chl) often display steep absorption gradients. This over-saturates photosynthesis in upper chloroplasts and deprives lower chloroplasts of blue and red light. Reducing chl content could create a more even leaf light distribution and thereby increase leaf light-use efficiency and overall canopy photosynthesis. This was tested on soybean cultivar ‘Clark’ (WT) and a near-isogenic chl *b* deficient mutant, *Y11y11*, grown in controlled environment chambers and in the field. Light attenuation was quantified using a novel approach involving light sheet microscopy. Leaf adaxial and abaxial surfaces were illuminated separately with blue, red, and green wavelengths, and chl fluorescence was detected orthogonally to the illumination plane. Relative fluorescence was significantly greater in deeper layers of the *Y11y11* mesophyll than in WT, with the greatest differences in blue, then red, and finally green light when illuminated from the adaxial surface. Modeled relative photosynthesis based on chlorophyll profiles and Beer’s Law predicted less steep gradients in mutant relative photosynthesis rates compared to WT. Although photosynthetic light-use efficiency was greater in the field-grown mutant with ~50% lower chl, light-use efficiency was lower in the mutant when grown in chambers where chl was ~80% reduced. This difference is probably due to pleiotropic effects of the mutation that accompany very severe reductions in chlorophyll and may warrant further testing in other low-chl lines.

## Highlight

Light sheet microscopy, a novel approach to quantifying light profiles, showed more gradual light attenuation in light-green soybean leaves compared to dark-green soybean.

## Introduction

A considerable amount of research has studied the light gradients within leaves. Because chlorophyll (chl) is packaged into discrete compartments (chloroplasts), the resulting ‘sieve effect’ reduces light absorption. Also known as absorption flattening, this phenomenon occurs in non-homogeneous distributions of chromophores because the effective molecular absorption cross-section decreases with concentration (Duyens, 1956). Conversely, increased path length due to light scattering, or the detour effect (Kok, 1948), occurs frequently in spongy mesophyll and increases light absorption. Thus, the decrease in absorption due to the sieve effect and the increase in absorption due to scattering have opposing dependencies on leaf chlorophyll content. Despite expected deviations from Beer’s Law presented by the sieve and detour effects, tissue-specific single-wavelength light attenuation follows Beer’s Law fairly well (Terashima and Saeki, 1983). As a result, wavelengths that are strongly absorbed by chl, such as red and blue light, are 90% attenuated in the upper 20% of the leaf (Cui *et al.*, 1991), leaving green light, for which chl has a much lower extinction coefficient, to drive a greater proportion of photosynthesis (*A*) in the lower leaf (Sun *et al.*, 1998; Terashima *et al.*, 2009).

Several methods have been employed for measuring leaf light profiles. These include optical property measurements on paradermal sections (Terashima and Saeki, 1983), microfiber optic measurements (Vogelmann *et al.*, 1991; Vogelmann, 1993), and chl fluorescence imaging (Takahashi, 1994; Vogelmann and Evans, 2002). The last mentioned method illuminates the sample from the adaxial or abaxial surface and views the fluorescence perpendicular to the illumination on a cut-edge cross-section (Vogelmann and Evans, 2002). A similar method involving light sheet microscopy (Weber *et al.*, 2014) could offer an alternative manner of measuring leaf chl fluorescence profiles. Light sheet microscopy is a fluorescence microscopy technique in which a sample is optically sectioned with a sheet of light, and since the detection is orthogonal to the illumination plane, there is no out-of-focus excitation or signal generated. In contrast, the illumination and detection planes are not separated in confocal microscopy, and the out-of-focus excitation is later removed by a pinhole in the emission path to obtain an optical section (Huisken *et al.*, 2004). By illuminating only the observation plane, light sheet microscopy also reduces photodamage and stress on the sample relative to other imaging techniques (Santi, 2011). Because light sheet microscopy sample illumination is perpendicular to the objective lens, leaf adaxial and abaxial surfaces may be illuminated at the same time or independently with detection on the cut edge, as in Vogelmann and Evans (2002), but with greater ease, precision, and resolution (<1 µm).

The light gradients present in leaves effectively alter photosynthetic capacity as a function of leaf depth. Chloroplasts in the upper and lower portions of the leaf acclimate to the gradient in light quantity and spectral distribution similar to sun and shade leaves, resulting in a decrease in photosynthetic capacity as chloroplasts become shaded (Terashima and Inoue, 1984, 1985*a*, *b*; Terashima and Hikosaka, 1995; Terashima *et al.*, 2005). The gradient of light also causes a gradient in photoprotection and thus a gradient in overall photosynthetic efficiency (Schreiber *et al.*, 1996; Oguchi *et al.*, 2011). Accordingly, greater photoprotection is found near the surface when illuminated with red or blue light and evenly dispersed throughout the leaf when illuminated with green light (Oguchi *et al.*, 2011). Measuring *A* profiles in leaves is difficult, but a multi-layer leaf model reasonably describes CO_2_ fixation profiles within C_3_ leaves as a function of either chl content and Beer’s Law or using ^14^C edge labeling techniques (Terashima and Saeki, 1985; Evans, 1995, 1999; Evans and Vogelmann, 2003). In addition, using chl content and Beer’s Law no longer requires labor-intensive paradermal sectioning and pigment extraction to determine chl profiles. Epi-illumination and detection of chl fluorescence of leaf cross-sections relates to chl distribution in leaf layers (Han *et al.*, 1999; Vogelmann and Evans, 2002) and can be used instead. A negative linear relationship exists between Rubisco per unit cumulative chl with depth from the adaxial surface (Terashima and Inoue, 1985*a*; Nishio *et al.*, 1993; Evans, 1995), which allows the estimation of Rubisco content, or effectively the maximum photosynthetic capacity for each leaf layer. Photosynthetic capacity combined with the amount of light absorbed by each leaf layer, which is determined using chl content and Beer’s Law, determine photosynthetic profiles (Evans, 1995) and provide insight into the distribution of *A* within leaves.

Reducing chl content through smaller antennae has been hypothesized to improve canopy light-use efficiency by creating a more even light distribution among leaves of a crop canopy (Ort *et al.*, 2011, 2015), but it may also alleviate the light disparity among chloroplasts within leaf layers. This could decrease efficiency losses caused by photoprotection at high light since upper chloroplasts would absorb less light. Additionally, more light would reach chloroplasts in the lower palisade or spongy mesophyll cells, resulting in greater leaf photosynthetic light-use efficiency. Support for this concept has been shown in light-green soybean (*Glycine max* Merr.) mutants with approximately one-third to one-half the chl of the dark-green WT. In these mutants, photosynthetic rates at the leaf level were often similar or greater compared to the WT when measured at the same light intensity (Xu *et al.*, 1993; Jiang *et al.*, 1997).

The primary focus of this study was to compare light absorption profiles within dark- and light-green soybean leaves grown in chamber and field settings using light sheet microscopy. Reducing chl was expected to reduce the disparity of light availability between the upper and lower chloroplasts of the leaf, reduce the modeled gradient of *A* across the leaf, and therefore explain the increases in light-green soybean mutant photosynthetic efficiency at the leaf level. Light sheet microscopy demonstrated the same patterns in leaf light attenuation in blue, red, and green light as previously reported. As expected, greater light absorption occurred in deeper leaf layers of the light-green mutant leaf with the most noticeable differences in blue light. In addition, modeled leaf *A* profiles based on chl profiles and Beer’s Law were also more gradual in the mutants. However, while photosynthetic light-use efficiency was greater in the field-grown mutant, it was lower in the mutant when grown in chambers, which may be due to confounding pleiotropic effects of the mutation.

## Methods

### Growth chamber experimental design

Soybean cultivar ‘Clark’ wildtype (WT) and a nearly isogenic chl deficient mutant, *Y11y11*, were grown in controlled environment growth chambers (model PCG20, Conviron, Winnipeg, Canada). Sample size was six for each genotype, where single plants represented biological replicates. Four *Y11y11* seeds (which segregate 1 dark-green; 2 light-green; 1 yellow lethal) were planted in each of 12 pots (7.6 l) filled with LC-1 Sunshine mix (SunGro Horticulture Canada Ltd, Bellevue, WA, USA) and thinned to one plant per pot of either WT or *Y11y11* after emergence. Growth conditions followed a 14h day/10h night cycle with daylight at approximately 700 µmol m^−2^ s^−1^, 65% relative humidity, and day/night temperatures of 25/22 °C. Beginning one week after emergence, plants were fertilized using 50% Long Ashton solution with an additional 10mM NH_4_NO_3_ (Hewitt, 1966) every other day and watered in between as needed.

### Field experimental design

The same genotypes were grown at the SoyFACE facility (40º02’N, 88º14’W, 228 m above sea level) at the University of Illinois at Urbana-Champaign during the 2014 growing season. Prior to planting on 17 June, fertilizer was applied according to standard procedure for corn-soy rotations in the Midwest, USA. Due to the segregation of *Y11y11* seeds, *Y11y11* was planted at double the density of WT. Shortly after emergence, all yellow plants died and all dark-green plants were removed, resulting in a similar density for each genotype (approximately 30 plants m^−2^). The experimental design consisted of a randomized complete block design (*n*=6) where an experimental unit consisted of a plot containing four 2.44 m rows running N–S with 0.38 m between rows.

### Light profiles

All measurements were conducted on sun leaves, which were designated as the youngest, fully expanded leaf on the main stem. All chamber-grown leaf tissue used for microscopy was removed from sun leaves still attached to the plant, whereas all field-grown leaf tissue was taken from leaves collected pre-dawn the same day. Light profiles were measured using light sheet microscopy (Lightsheet z.1, Zeiss, Obercohen, Germany; see Supplementary Fig. S1 at *JXB* online for a diagram of the sample positioning and illumination). Leaf samples approximately 1–2mm wide and 15–20mm long were cut from interveinal regions. The sections were then embedded in 1% low-melting and low-gelling agarose within a glass capillary with an inner diameter of 2.15mm. Once the agarose had solidified, the sample was partially ejected into the sample turret and suspended in water so that the adaxial and abaxial surfaces were perpendicular to the light sheet illumination objective (LSFM 10×/0.2 NA; Zeiss, Obercohen, Germany) and the cut edge was facing the detection objective (W Plan-Apochromat 20×/1.0 NA; Zeiss, Obercohen, Germany). At non-saturating intensities, excitation alternated between the adaxial and abaxial surfaces at wavelengths of 445, 638, and 561nm for the chamber-grown plants. The wavelengths used in the field experiment consisted of 405, 488, 638, and 561nm. A long pass filter was used to collect chl fluorescence above 660nm. The thickness of the light sheet was 4.8 µm, and pixel size was 0.23×0.23 µm. A *z*-stack of at least 25 µm and consisting of approximately 40–45 complete leaf cross-section images was collected at two locations per leaf sample in the chamber experiment and at three locations per leaf in the field experiment for technical replication.

Images were analyzed using Zen software (Blue Edition; Zeiss, Obercohen, Germany). A 200-µm long cross-section was analyzed where mean intensity from the illuminated leaf surface was recorded at 0.23 µm intervals. Sections were lined up along the outermost edge of the leaf according to the direction of illumination. Three different cross-sections in the *z* direction (depth from cut surface) per technical replication were analyzed as subsamples, which were approximately 5 µm apart to ensure sections were from non-overlapping light sheets. Means and standard deviations were calculated after averaging all subsamples by pixel layer from the illuminated surface. Relative fluorescence was then calculated by dividing by the maximum fluorescence for each wavelength and genotype. The same scalars were applied to the standard deviations, which were then divided by the square root of *n* (*n*=6) to obtain standard errors. Paired *t*-tests were conducted at each pixel layer, and significant differences in light absorbance were determined at alpha =0.05.

### Sampling

Leaf absorbance of visible light wavelengths was measured using an integrating sphere (Spectroclip-JAZ-TR, Ocean Optics, Duiven, The Netherlands) on the adaxial surface in both experiments and the abaxial surface in the field experiment. The percent leaf absorbance was determined as

$$\%Abs=\;\left(I_{\text{o}}-I_{\text{r}}-I_{\text{t}}\right)\times 100$$

where *I*_o_ is incident light, *I*_r_ is reflected light, and *I*_t_ is transmitted light. Path lengthening was determined for both genotypes using Beer’s Law to calculate the ratio of expected absorbance to actual absorbance. Expected absorbance was calculated as

$$Abs_{\text{1}}=\varepsilon cl$$

where *ε* is the extinction coefficient of chl in pigment–protein complexes [2230 m^2^ (mol chl) ^−1^; Evans, 1995], *c* is the concentration of chl in the leaf (mol m^−3^), and *l* is the leaf thickness determined from light sheet images. Actual absorbance was calculated by

$$Abs_{\text{2}}=\text{ log}\left(P_{\text{o}}/P\right)$$

where *P*_o_=100% and *P*=(100% – *%Abs*). The apparent *ε* was then calculated by substituting *Abs*_2_ for *Abs*_1_ in the expected absorbance equation and rearranging the equation to

$$\varepsilon =Abs_{\text{2}}/cl$$

Chl was extracted from 1-cm diameter leaf disks to determine total chl content and chl *a*/*b* ratios according to Porra *et al.* (1989) and Lichtenthaler (1987). Specific leaf weight (SLW) was determined from the mass of a 2-cm leaf disk after drying to constant weight. Leaf tissue was also collected from field-grown plants for Rubisco quantification. Tissue was ground in liquid nitrogen, and approximately 100mg was added to extraction buffer and centrifuged to remove solid matter. Total protein concentration was determined using a Bradford assay, and 2 µg total protein was loaded from each sample for gel electrophoresis. Protein was then blotted onto a polyvinylidene difluoride (PVDF) membrane and treated with a primary antibody for the large subunit of Rubisco, and then a secondary antibody. The membrane was imaged by chemiluminescence (SuperSignal West Femto, Pierce Biotechnology, Rockford, IL, USA) using a blot scanner (C-DiGit Blot Scanner, LI-COR, Lincoln, NE, USA), and band intensity was quantified from the image (Image Studio 5.0 Software, LI-COR, Lincoln, NE, USA). Relative chl content profiles as a function of depth into the leaf were determined using epi-fluorescence and detection in the same manner as Vogelmann and Evans (2002). Confocal microscopy (LSM710, Zeiss, Obercohen, Germany) was used to illuminate and detect fluorescence from the cut surface so that all layers of the leaf cross-section were illuminated evenly, as opposed to light sheet microscopy where the light intensity was greatest at the leaf surface nearest the illumination objective. Leaf sections 5×10mm were embedded in 1% ultra-pure agarose and finely sliced into cross-sections. The cut edge was illuminated with 488nm light with the resulting chl fluorescence detected at 679nm. Cross-section fluorescence from adaxial to abaxial surface over a 200-µm leaf section was quantified in Zen software (Blue Edition; Zeiss, Obercohen, Germany), normalized for leaf depth, and analyzed in the same manner as light sheet microscopy data.

Analyses of variance on chl *a*/*b*, chl content, carotenoid content, *%Abs*, path length, SLW, and relative Rubisco were conducted in Proc GLM (SAS 9.4, SAS Institute, Cary, NC, USA) with genotype considered a fixed effect. Means were based on *n*=6. Differences were considered significant at alpha =0.05.

### Modeling

Epi-fluorescence, which proportionally represented relative chl content, was converted to actual chl content based on whole-leaf chl contents (see Results). Rubisco content per unit chl has a negative linear relationship with cumulative chl content (Evans, 1995). Soybean leaves are relatively thin compared to spinach leaves, making it extremely difficult to obtain multiple paradermal sections for Rubisco analyses. Therefore, it was assumed that the same relative decline in Rubisco with cumulative chl content occurs in soybean as it does in spinach. Using this relationship, Rubisco content was calculated for each layer, after which Rubisco per chl was multiplied by chl content and divided by the greatest Rubisco content to calculate relative Rubisco content per layer in each genotype. Absorbance was calculated as *Abs*_1_ = *εcl* for each layer based on the apparent *ε* calculated above. Using *Abs*_1_ in place of *Abs*_2_ in the equation *Abs*_2_ = log(*P*_o_/*P*), *P* was calculated to determine the amount of light absorbed (*I*_a_) and available in each layer [based on 2000 µmol m^−2^ s^−1^ incident photosynthetic photon flux density (PPFD) at the adaxial surface]. *A* by layer (*A*_i_) was calculated using the multi-layer leaf model (Terashima and Saeki, 1985; Evans, 1995, 1999; Evans and Vogelmann, 2003):

$$A_{\text{i}}=\;(\phi I_{\text{a}}+A_{\text{m}}-\;{\left[{\left(\phi I_{\text{a}}+A_{\text{m}}\right)}^{\text{2}}-\text{ 4}\theta \phi I_{\text{a}}A_{\text{m}}\right)}^{0.\text{5}}]/\text{2}\theta$$

where *ϕ*, representing *ϕCO*_*2*_, and *θ*, the curvature factor, were estimated from light response curves (see below) and assumed to be the same for each layer (Evans, 1995), maximum photosynthetic capacity (*A*_m_) was based on relative Rubisco content, and absorbed light per layer (*I*_a_) was calculated as above. *A*_i_ was then converted to relative *A*_i_ by dividing all layers by the maximum calculated *A*_i_ for each genotype and experiment. All modeling was based on relative distance from the adaxial surface of the leaf due to slight variation in leaf thickness between growth environments. Leaf thickness was slightly lower in the field compared to the growth chamber experiment but did not differ between genotypes (data not shown).

### Gas exchange

All gas exchange measurements on chamber plants were conducted prior to light sheet microscopy measurements and on the same leaves as the light sheet measurements. Field gas exchange occurred within two days following microscopy on different plants within plots but at the same relative leaf position in the canopy. Photosynthetic light (*A*/*Q*) and CO_2_ (*A*/*C*_i_) response curves were measured using an open-path gas exchange system equipped with a leaf chamber fluorometer (LI-6400, LI-COR, Lincoln, NE, USA). On the same day as *A*/*Q* curve measurements, dark-adapted minimal (*F*_o_) and maximal fluorescence (*F*_m_) of photosystem II (PSII) were measured prior to growth chamber illumination or dawn. This allowed determination of maximum efficiency of PSII in the light (*F*_v_′*/F*_m_′), non-photochemical quenching [*NPQ*, (1–*F*_*m*_′)/*F*_*m*_′), and photochemical quenching factor [*q*_P_ = (*F*_m_′-*F*_s_′)/*F*_v_′, where *F*_s_ *′* is steady-state fluorescence] as a function of irradiance. The operating efficiency of PSII [*ϕPSII* = (*F*_*m*_′- *F*_*s*_′)/*F*_*m*_′] was also measured at each irradiance. Incident PPFD was adjusted for absorption after determining leaf absorbance in the red and blue wavelengths emitted by the fluorometer LEDs. Saturated rates of *A* (*A*_sat_), maximum quantum efficiency (*ϕCO*_*2*_), response curvature (*θ*), and dark respiration rates (*R*_d_) versus absorbed PPFD were determined by fitting the data to a non-rectangular response curve (SigmaPlot, Systat Software Inc, San Jose, CA, USA). Proc Loess (SAS 9.4; SAS Institute, Cary, NC, USA) was used to determine 95% confidence intervals for all *A*/*Q* data where non-overlapping intervals indicated significant differences. *A*/*C*_i_ curves were analyzed to determine the maximum carboxylation capacity of Rubisco (*V*_c,max_), the maximum rate of electron transport (*J*_max_) and the intercellular concentration of CO_2_ (*C*_i_) at the inflection point of the curve (*C*_i,inflection_) according to Long and Bernacchi (2003). Analyses of variance on photosynthetic parameters (*V*_c,max_, *J*_max_, *C*_i,inflection_, *A*_sat_, *ϕCO*_*2*_, *θ*, *R*_d_, *F*_v_/*F*_m_) were conducted in Proc GLM (SAS 9.4, SAS Institute, Cary, NC, USA) with genotype considered as a fixed effect. Means were based on *n*=6 except for field *A*/*C*_i_ parameters (*n*=5), field *A*/*Q* gas exchange parameters (*n*=4), and chamber *F*_v_/*F*_m_ (*n*=4). Differences were considered significant at alpha =0.05.

## Results and Discussion

### Reducing chl content significantly affected light attenuation, especially in blue and red wavelengths

**Fig. 1.**
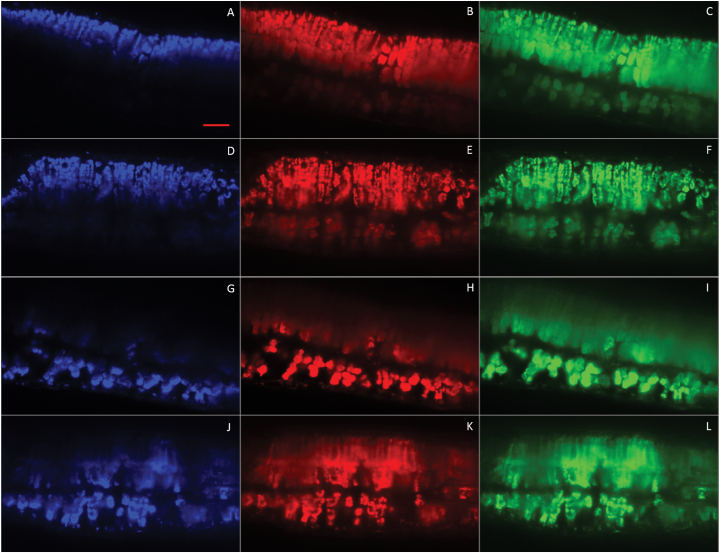
Fluorescence profiles of chamber-grown WT (ABCGHI) and *Y11y11* (DEFJKL) cross-sections within a *z*-stack when illuminated from the adaxial (A–F) or abaxial (G–L) surface with blue (445nm; A, D, G, J), red (638nm; B, E, H, K), and green (561nm; C, F, I, L) lasers using light sheet microscopy. Fluorescence was falsely colored to represent the illumination wavelength. Scale bar in (A) = 50 µm. Pixel size = 0.23 µm.

Fluorescence profiles from light sheet microscopy resembled previous leaf profile measurements on other species using monochromatic light perpendicular to the leaf surface. As expected, blue light (445nm) was most sharply attenuated as a function of depth within the leaf in both genotypes, regardless of illumination direction (Fig. 1A, D, G, J). As was seen in spinach leaves (Vogelmann and Evans, 2002), the attenuation of red light (638nm) was somewhat more gradual (Fig. 1B, H), and green light (561nm) attenuation was the most gradual (Fig. 1C, I) in WT leaves. In *Y11y11* leaves, red light attenuation was similar to green light attenuation (Fig. 1E, F, K, L). The profiles also demonstrated a clear distinction between palisade and spongy mesophyll (Fig. 1), which was evident in the ‘shoulders’ especially visible in red and green light fluorescence quantification (see below). These profiles, especially in the WT soybean leaves, were very similar to those shown by Vogelmann and Evans (2002), although the resolution depicted here was greater as pixel size was reduced to 0.23×0.23 µm.

**Fig. 2.**
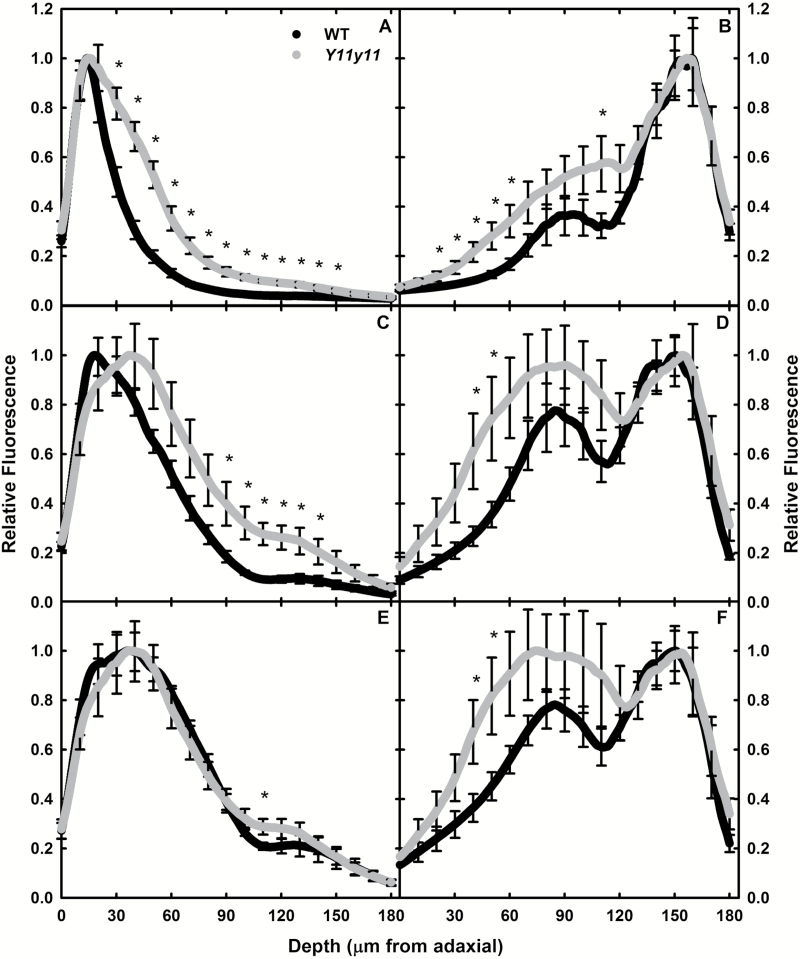
Relative fluorescence with distance from the adaxial surface. WT (black) and *Y11y11* (grey) chamber-grown leaves were illuminated with blue (445nm; A, B), red (638nm; C, D), and green (561nm; E, F) lasers from the adaxial (A, C, E) and abaxial (B, D, F) surface using light sheet microscopy. Mean fluorescence (*n*=6) is shown every 0.23 µm. Error bars are indicated every 10 µm. Asterisks represent significant differences at *P*<0.05.

**Fig. 3.**
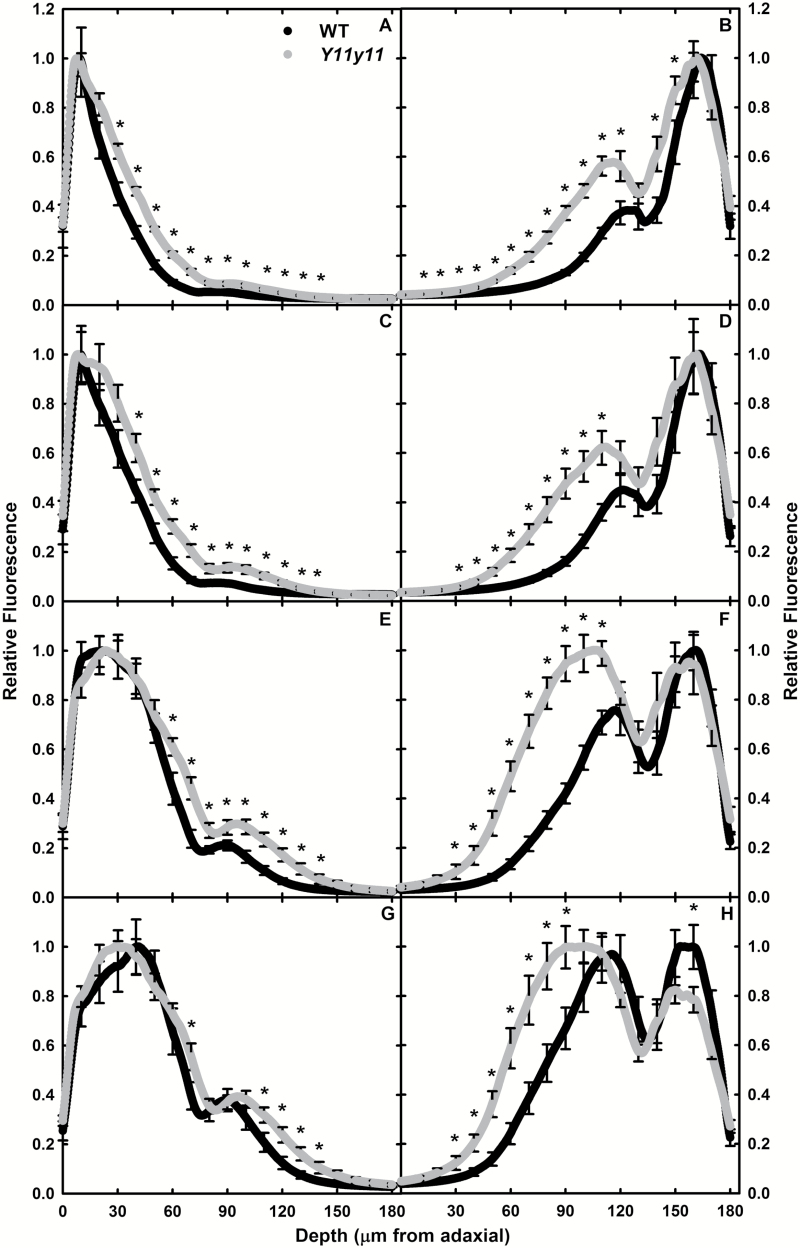
Relative fluorescence with distance from the adaxial surface. WT (black) and *Y11y11* (grey) field-grown leaves were illuminated with 405nm (A, B), 488nm (C, D), 638nm (E, F), and 561nm (G, H) lasers from the adaxial (A, C, E, G) and abaxial (B, D, F, H) surface using light sheet microscopy. Means (*n*=6) are shown every 0.23 µm. Error bars are indicated every 10 µm. Asterisks represent significant differences at *P*<0.05.

When illuminating the leaf samples from the adaxial surface, blue light peak absorbance occurred at approximately the same location in the leaf for both genotypes (15 µm from the adaxial surface in chamber plants and 8–10 µm for field plants), but the mutant leaf had significantly greater blue light absorbance at greater depths in the leaf (Figs 2A, 3A, C). Very little blue light reached the spongy mesophyll in either leaf, as demonstrated by lack of a clear ‘shoulder’ in both profiles (Figs 2A, 3A, C) that was evident in the red and green adaxial illumination (Figs 2C, E, 3E, G). The ratio of the blue-to-red molar absorption coefficient is much higher for chl *b* than for chl *a*, and thus explains blue light attenuation before reaching the spongy mesophyll. In addition, the difference in red and blue absorptivity could have contributed to the deeper penetration of blue light into the *Y11y11* leaf that is largely devoid of chl *b* (Table 1). However, in both experiments, significantly more red light was also absorbed in the lower palisade and upper spongy mesophyll cells in the mutant compared to the WT (Figs 2C, 3E). In the chamber-grown plants, peak absorbance in red light occurred much deeper in the *Y11y11* leaf (37 µm from the adaxial surface) as compared to the WT (17 µm; Fig. 2C). In field-grown leaves, the peak of red light absorption was approximately 21–23 µm in both genotypes (Fig. 3E). Green light peak absorbance occurred at approximately the same location in all genotypes across experiments (30–40 µm; Figs 2E, 3G). Only slightly more green light was available in spongy mesophyll cells of the mutant compared to WT (Figs 2E, 3G). These data supported the hypothesis that reducing pigment concentrations facilitates a more even light distribution of highly absorbed wavelengths in the light-green leaf, resulting in proportionally more light absorption in the mutant spongy mesophyll as compared to the palisade mesophyll. Moreover, the change was not limited to a deficiency in chl *b* due to similar patterns in blue and red light.

**Table 1.** Summary of leaf properties in chamber- and field-grown dark-green WT and light-green *Y11y11* ‘Clark’ soybean. Means of chl *a*/*b* ratios, total chl content, leaf absorbance (*%Abs*), calculated path length, and specific leaf weight (SLW) are reported with the ratio of the chl-deficient mutant as compared to the WT. ANOVA summary statistics are also indicated (*n*=6)

Parameter	WT	*Y11y11*	Ratio	*F*-value	*P*-value
*Chamber experiment*					
Chl *a*/*b* ratio	2.92	4.51	1.5	502	<0.0001
Chl (µmol m^−2^)	301.3	61.8	0.21	1414	<0.0001
Carotenoids (g m^−2^)	41.7	19.2	0.46	381	<0.0001
*%Abs*	92.2	67.5	0.73	992	<0.0001
Path lengthening	1.66	3.57	2.15	191	<0.0001
SLW (g m^−2^)	23.2	18.0	0.78	51.5	<0.0001
*Field experiment*					
Chl *a*/*b* ratio	3.09	4.60	1.5	197	<0.0001
Chl (µmol m^−2^)	457	212	0.46	274	<0.0001
Carotenoids (g m^−2^)	72.5	53.0	0.73	39.7	<0.0001
*%Abs* (from adaxial)	93.2	86.3	0.93	43.7	<0.0001
*%Abs* (from abaxial)	90.6	82.3	0.91	102	<0.0001
Path lengthening	1.15	1.84	1.60	41.5	<0.0001
SLW (g m^−2^)	46.4	45.2	0.97	0.61	0.45
Rubisco (relative units)	0.778	0.745	0.96	0.14	0.71

**Fig. 4.**
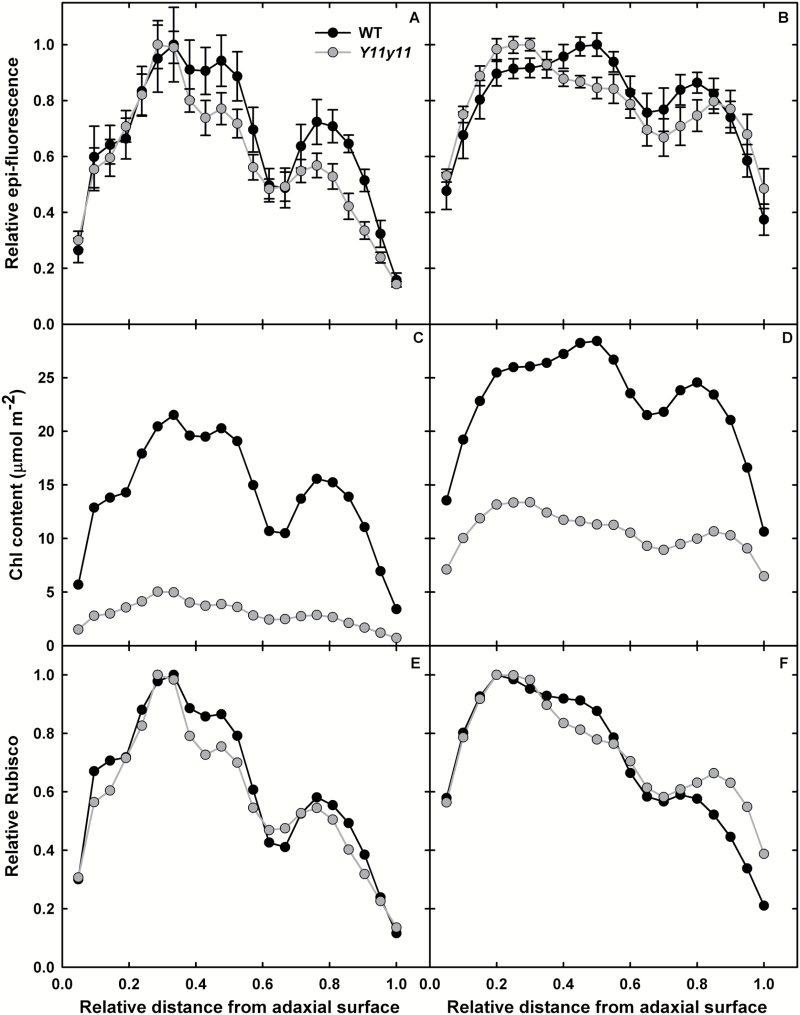
Epi-fluorescence, chl content, and relative Rubisco profiles in chamber-grown (A, C, E) and field-grown (B, D, F) WT (black) and *Y11y11* (grey) leaves. Relative chl distribution from epi-fluorescence measurements (A, B) was converted to absolute chl content (C, D) based on total leaf chl content on an area basis. Relative Rubisco (E, F) was determined from previous relationships between Rubisco per chl versus cumulative chl (Evans, 1995, 1999). Sample size in (A, B) was *n*=6 except for chamber-grown *Y11y11* (*n*=5). Error bars represent standard errors of the means.

When illuminated from the abaxial surface, significant differences between genotypes were more common in the field experiment. Chamber-grown WT and *Y11y11* fluorescence profiles were similar in the spongy mesophyll within each illumination wavelength, with limited significant differences in fluorescence occurring in the palisade mesophyll (Fig. 2B, D, F), whereas field-grown *Y11y11* plants demonstrated significantly greater relative fluorescence in the palisade mesophyll cells across all wavelengths (Fig. 3B, D, F, H), greater fluorescence in spongy cells with blue light (405nm; Fig. 3B), and significantly less fluorescence in spongy cells with green light (561nm; Fig. 3H). Genotypic differences in the location of peak absorbance within experiments only occurred in red and green wavelengths. Peak absorbance of red light was again similar in WT and *Y11y11* chamber plants (26–30 µm; Fig. 2D), but in field plants *Y11y11* peak absorbance was much further from the abaxial surface (72 µm) as compared to the WT (18 µm; Fig. 3F). Peak absorbance of green light occurred in the palisade mesophyll of *Y11y11* (105 µm from the abaxial surface in chamber plants and 78 µm in field plants; Figs 2F, 3H) but occurred in the spongy mesophyll of WT leaves (25–30 µm; Figs 2F, 3H). This may have been due to chl distribution patterns differing between WT and *Y11y11* leaves in chamber versus field conditions. In the chamber-grown plants, WT and *Y11y11* relative chl content peaked in the upper palisade mesophyll (Fig. 4C). The same was true for the field-grown mutant, but the field-grown WT relative chl content peak occurred in the lower palisade (Fig. 4D).

### Leaf properties were significantly altered by chl reductions

At the time of measurement, the contrast between WT and *Y11y11* leaf properties was much greater in chamber-grown soybean plants compared to field-grown plants. Although chl *a*/*b* ratios were 1.5 times greater in *Y11y11* compared to WT in both experiments (*P*<0.0001; Table 1), overall chl content was reduced by almost 80% in chamber-grown mutants (*P*<0.0001; Table 1) as compared to a 54% reduction in field-grown mutants compared to WT (*P*<0.0001; Table 1). Carotenoid content was also reduced by 54% in chamber-grown *Y11y11* leaves (*P*<0.0001; Table 1) as opposed to a 27% decrease in field-grown *Y11y11* leaves compared to WT (*P*<0.0001; Table 1). *Y11y11* SLW was reduced by 20% of the WT in the chamber experiment (*P*<0.0001; Table 1), but the reduction in field-grown mutant plants was only 3% (*P*=0.45; Table 1). Previous experiments have shown variability in the extent of the *Y11y11* phenotype, mainly as a function of light intensity (Xu *et al.*, 1993). Light availability was probably the cause of the phenotypic differences in this study since light intensity inside the growth chamber (700 µmol m^−2^ s^−1^ PPFD) was significantly lower than the full sunlight experienced by the plants grown in the field (approximately 2000 µmol m^−2^ s^−1^ PPFD).

Reductions in *%Abs* were disproportionately less than reductions in chl content in both experiments and lower than predicted by Beer’s Law. Although chl content was reduced by 80% in the chamber experiment, *%Abs* was only reduced by approximately 25% (Table 1). In the field experiment, a >50% reduction in chl content only resulted in a 7% reduction in *%Abs* when illuminated from the adaxial surface and 9% when from the abaxial surface (Table 1). Comparisons between *Abs*_1_ and *Abs*_2_ revealed a 2.15 times greater path length in the mutant compared to the WT in the chamber experiment (*P*<0.0001; Table 1) and a 1.6 times greater path length in the mutant in the field experiment (*P*<0.0001; Table 1). These values agreed with the relationship between chl reductions and path lengthening in low-chl plants compared to full-green plants calculated from McClendon and Fukshansky (1990; Supplementary Fig. S2). Cell shape and intercellular air-cell wall interfaces affect light scattering with the irregularly shaped spongy mesophyll linked to higher light scattering and therefore greater path lengthening compared to the columnar-shaped palisade mesophyll cells (Vogelmann, 1993). With reduced chl, less light was expected to be absorbed in the upper leaf, allowing more light to reach the spongy mesophyll where it would be scattered and therefore have a greater chance of absorption before exiting the leaf as transmitted light. This effect would increase the path length to a greater degree in the mutant as compared to the WT. When illuminating from the abaxial side, overall *%Abs* decreased in both genotypes (Table 1), which was most likely due to greater reflectance with the increased scattering of light in the spongy mesophyll as opposed to the focusing effect of columnar palisade cells (Terashima and Saeki, 1983; Vogelmann, 1993).

### Modeled photosynthetic profiles predict a more even distribution of carbon capture across mutant leaf layers

Total leaf chl content (Table 1) and epi-fluorescence (Fig. 4A, B) were used to estimate leaf chl content profiles (Fig. 4C, D). Relative epi-fluorescence patterns were similar in chamber-grown WT and *Y11y11* leaves but with slightly greater values in WT lower palisade and spongy mesophyll tissue (Fig. 4A). A similar relationship was evident between field-grown WT and *Y11y11* leaves (Fig. 4B), but peak WT fluorescence occurred at a greater depth as compared to *Y11y11* (Fig. 4B). Since chl content is proportional to fluorescence (Han *et al.*, 1999; Vogelmann and Evans, 2002), the chl profiles demonstrated the same patterns as epi-fluorescence adjusted for total chl content (Fig. 4C, D). Total leaf Rubisco content was the same in WT and *Y11y11* field-grown plants (Table 1, Supplementary Fig. S3). Using the equations previously relating Rubisco to chl content (Terashima and Inoue, 1985*a*; Nishio *et al.*, 1993; Evans, 1995) and assuming a similar relationship applies in soybean, Rubisco content as a function of cumulative chl content was first calculated and then used to determine relative Rubisco as a function of relative depth within the leaf (Fig. 4E, F) to estimate *A*_m_ for each leaf layer. Relative Rubisco distribution was similar between experiments (Fig. 4E, F), with the notable exception of greater relative Rubisco content in field-grown mutant spongy mesophyll (Fig. 4F).

**Fig. 5.**
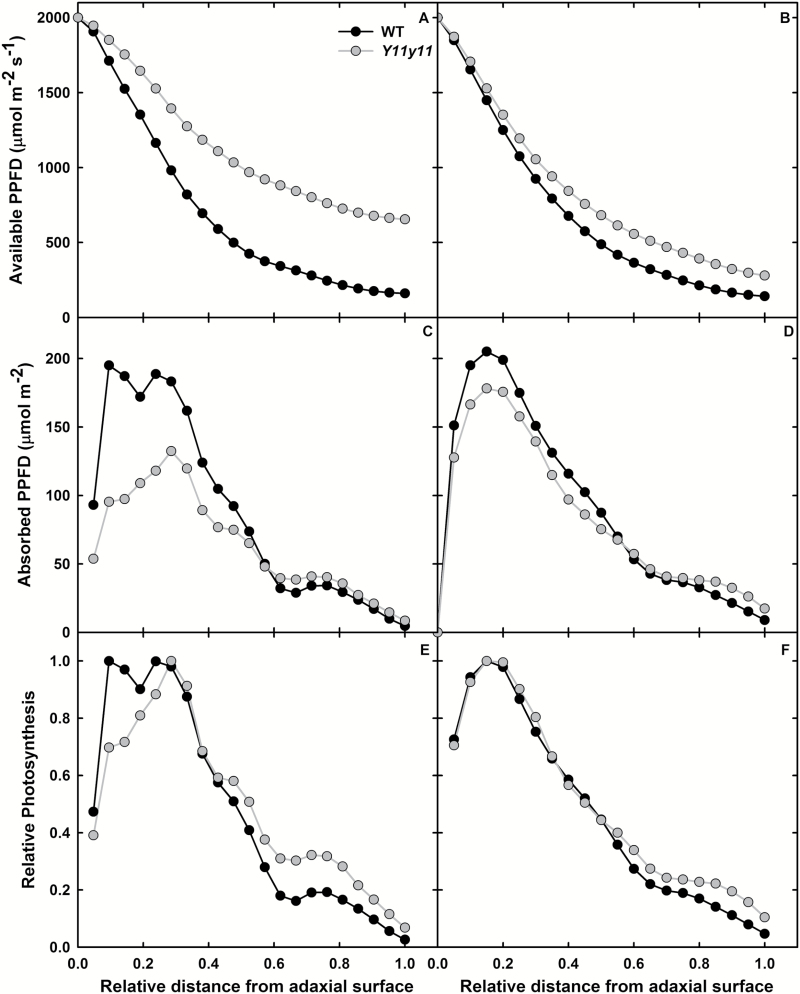
Light availability, absorption, and relative photosynthesis profiles in chamber-grown (A, C, E) and field-grown (B, D, F) WT (black) and *Y11y11* (grey) leaves. The amount of light available (A, B) and absorbed (C, D) in each layer were used to determine relative photosynthesis profiles (E, F).

Based on Beer’s Law and chl content profiles, light availability by layer was greater with depth in the *Y11y11* leaf in both experiments (Fig. 5A, B) with a greater difference between WT and *Y11y11* in the chamber experiment (Fig. 5A). WT light absorption near the adaxial surface was greater than that of *Y11y11* (Fig. 5C, D), which most likely drove differences in total leaf absorbance (Table 1). However, *Y11y11* light absorption was slightly greater in spongy mesophyll tissues in both experiments (Fig. 5C, D) due to greater light availability with depth in the mutant leaf (Fig. 5A, B). The difference between *Y11y11* and WT absorption in the lower leaf (Fig. 5C, D) was probably small because of very low chl content in the *Y11y11* spongy mesophyll (Fig. 4A, B). Relative *A*_i_ was greater in the mutant versus WT in lower leaf layers (Fig. 5E, F), therefore demonstrating more evenly distributed rates of *A*_i_ across the light-green leaf layers compared to WT. However, absorbed light was determined using the apparent *ε* of the whole leaf. Correcting light absorption for a layer-specific path lengthening or apparent *ε*, which would be greater in the spongy mesophyll, combined with greater light availability in *Y11y11* (Fig. 5A, B) would have probably increased overall light absorption to a greater extent in lower leaf layers of the mutant compared to the WT. If so, *Y11y11* would demonstrate even greater relative rates of *A*_i_ and therefore a smaller disparity between upper and lower leaf CO_2_ fixation.

### Despite more evenly distributed light and CO_2_ fixation in both experiments, only field-grown mutants demonstrated greater photosynthetic capacity and light-use efficiency

**Fig. 6.**
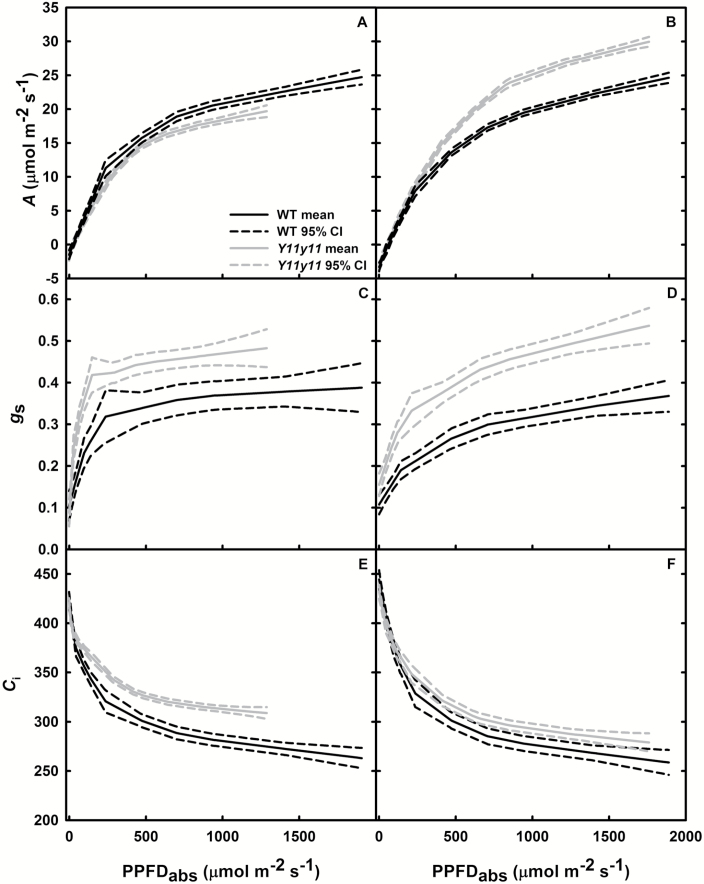
Gas exchange from light response curves. *A* (A, B), *g*_s_ (C, D), and *C*_i_ (E, F) were measured as a function of absorbed photosynthetic photon flux density (PPFD_abs_) in WT (black) and *Y11y11* (grey) chamber-grown (A, C, E) and field-grown (B, D, F) soybean. Solid lines represent the mean and dashed lines represent the 95% confidence intervals (*n*=6 in A, C, E; *n*=4 in B, D, F).

Although both chamber- and field-grown mutant soybean leaves demonstrated relatively greater light absorption (Figs 2, 3, 5A, B) and greater relative *A*_i_ in the lower leaf (Fig. 5E, F), chamber-grown mutants showed significant reductions in many *A*/*C*_i_ and *A*/*Q* parameters indicative of photosynthetic capacity (Table 2). *V*_*c,max*_ and *J*_max_ were approximately 25% lower in the mutant as compared to the WT (*P*<0.001; Table 2), suggesting a significant reduction in Rubisco content and photosynthetic machinery. At high light, carboxylation capacity limits *A* (Ogren and Evans, 1993), and this was evident in a 21% decrease in *Y11y11 A*_sat_ (Table 2). Light-use efficiency also declined in the mutant, as evident by lower rates of *A* versus absorbed PPFD at both high and low light levels (Fig. 6A). The maximum quantum efficiency at low light, or *ϕCO*_*2*_, was significantly reduced by 21% in the mutant (Table 2). Large, interconnected light-harvesting complexes associated with PSII (LHCII) decrease the chances of an absorbed photon being lost as thermal dissipation or fluorescence before reaching an open reaction center (Allen and Forsberg, 2001). Therefore, severely truncated LHCII in the chamber-grown mutants probably reduced *ϕCO*_*2*_ through inhibiting excitation transfer among photosystems when light was limiting (Zhu *et al.*, 2010), although a greater ratio of PSII to photosystem I (PSI) ratio in the mutant (Ghirardi and Melis, 1988) may have also been a contributing factor.

**Table 2.** Summary of gas exchange and dark-adapted fluorescence measurements for chamber- and field-grown dark-green WT and light-green Y11y11 ‘Clark’ soybean. Means of light response parameters, CO_2_ response parameters, and dark-adapted fluorescence measurements are reported with the ratio of the chl-deficient mutant as compared to the WT. ANOVA summary statistics are also indicated where n=6 for all parameters except chamber F_v_/F_m_ (n=4), field *A*/*Q* (n=4), and field *A*/*C*_i_ parameters (*n*=5)

	Parameter	WT	*Y11y11*	Ratio	*F*-value	*P*-value
*Chamber experiment*						
*A*/*Q*	*A*_sat_	28.3	22.3	0.79	32.4	<0.001
	*ϕCO*_*2*_	0.068	0.053	0.79	18.2	<0.01
	*R*_d_	1.69	1.43	0.82	4.89	0.052
	*θ*	0.582	0.798	1.40	4.56	0.059
*A*/*C*_*i*_	*V*_c,max_	106.6	79.8	0.75	21.2	<0.001
	*J*_max_	192.5	140.6	0.73	21.5	<0.001
	*C*_i,inflection_	286.8	260.5	0.91	6.20	<0.05
Fluorescence	*F*_v_/*F*_m_	0.824	0.824	1.00	<0.01	0.97
*Field experiment*						
*A*/*Q*	*A*_sat_	32.6	38.4	1.18	9.02	<0.05
	*ϕCO*_*2*_	0.062	0.061	0.98	0.09	0.77
	*R*_d_	0.44	0.63	1.43	9.10	<0.05
	*θ*	3.42	3.26	0.95	0.24	0.64
*A*/*C*_*i*_	*V*_c,max_	113.7	121.8	1.07	2.13	0.18
	*J*_max_	176.7	195.8	1.11	5.21	0.052
	*C*_i,inflection_	202.0	209.0	1.03	0.11	0.75
Fluorescence	*F*_v_/*F*_m_	0.75	0.74	0.99	0.04	0.85

While photosynthetic capacity and quantum efficiency were inhibited with the severe reductions in chl of chamber-grown plants, the field-grown chl reductions of 55% improved *Y11y11 A*_sat_ by 18% (*P*<0.05; Table 2) and did not significantly inhibit any other parameters (Table 2). Additionally, *Y11y11* field plants demonstrated greater photosynthetic light-use efficiency at mid- to high light levels, as demonstrated by greater photosynthetic rates per absorbed photon (Fig. 6B). This may in part have been due to greater *g*_s_ in the mutant (Fig. 6C, D), which may be caused by a mutation in the gene encoding magnesium chelatase subunit-I (*CHLI*; Campbell *et al.* 2015) that results in ABA insensitivity (Tsuzuki *et al.*, 2011; Du *et al.*, 2012) independently of chl concentration (Du *et al.*, 2012). Greater *g*_s_ increases CO_2_ availability or *C*_i_, which was greater in the mutant compared to WT in both experiments (Fig. 6E, F). Although the increase in *Y11y11 C*_i_ compared to WT *C*_i_ occurred to a lesser extent in the field experiment (Fig. 6F) than the chamber experiment (Fig. 6E), it, along with differences in photosystem stoichiometry, still represents confounding effects on leaf photosynthetic capacity that deserves further examination in mutants without these pleiotropic effects.

**Fig. 7.**
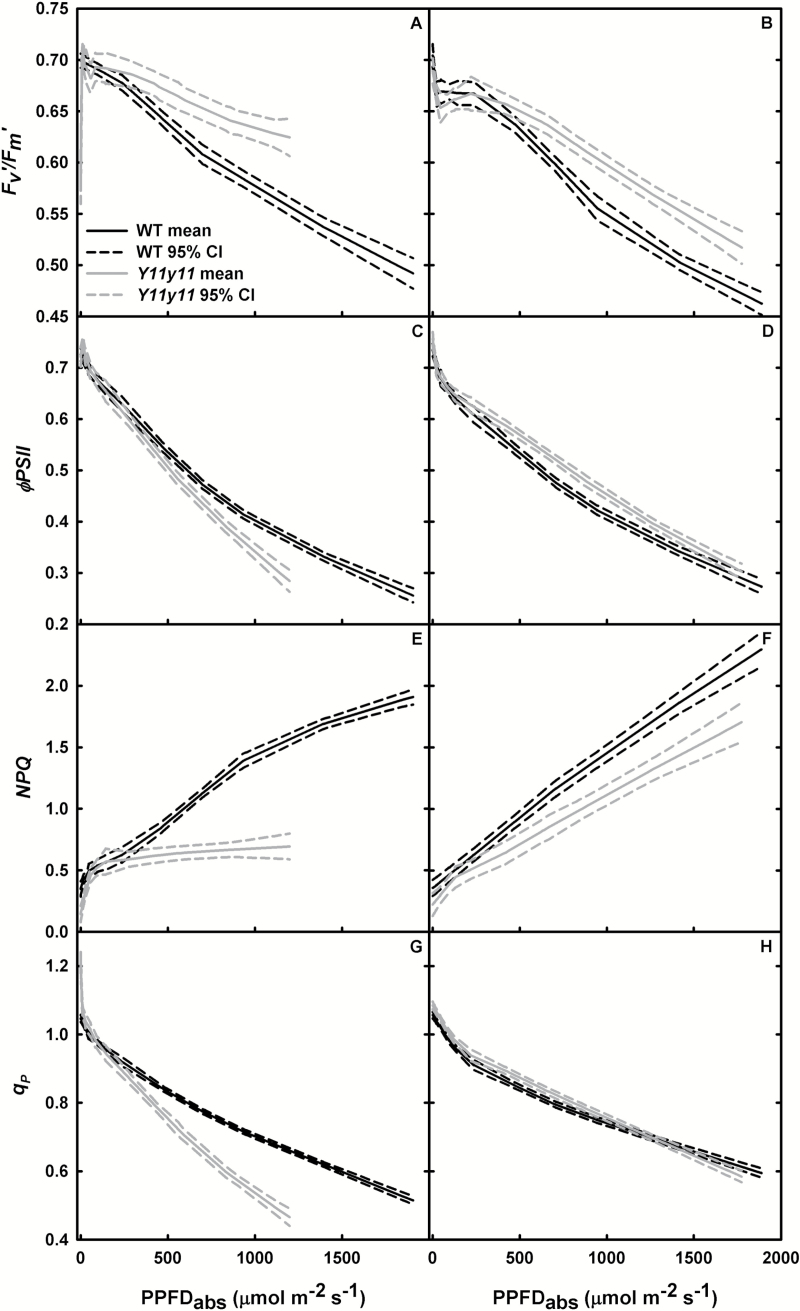
Chl fluorescence parameters from light response curves. *F*_v_′/*F*_m_′ (A, B), *ϕPSII* (C, D), *NPQ* (E, F), and *q*_P_ (G, H) were measured as a function of absorbed photosynthetic photon flux density (PPFD_abs_) in WT (black) and *Y11y11* (grey) chamber-grown (A, C, E, G) and field-grown (B, D, F, H) soybean. Solid lines represent the mean and dashed lines represent the 95% confidence intervals (*n*=6).

Photosynthetic and photoprotective performance, as indicated by modulated chl fluorescence measurements, also varied by experiment. Both chl fluorescence parameters were reduced when chl content was reduced by ~80% in the chamber-grown *Y11y11* mutant. Dark adapted *F*_v_/*F*_m_ was not significantly altered by genotype in either experiment (Table 2), indicating no severe photodamage caused by the chl mutation. However, chamber-grown mutants exhibited lower *ϕPSII* and *q*_P_ at higher light levels (Fig. 7C, G). *ϕPSII* is affected by end-product utilization. Accumulation of NADPH reduces the efficiency of Q_A_ oxidation (decreased *F*_q_/*F*_v_ or *q*_P_) through lower linear electron flux, and a higher ATP/ADP ratio causes acidification of the lumen, which in turn decreases *F*_v_′/*F*_m_′ as *NPQ* increases (Baker, 2008). Build-up of NADPH and ATP in high light can occur for a variety of reasons, including decreased carboxylation capacity as was evident in chamber-grown *Y11y11* (Table 2). This was coupled with a lower *q*_P_ in *Y11y11* at high light (Fig. 5E), indicating that limited Q_A_ oxidation (*q*_P_) was driving the decrease in *ϕPSII* (Fig. 5C). However, the negative effects of end-product build-up were not evident in chamber *Y11y11 F*_v_′/*F*_m_′ and *NPQ* measures at high light (Fig. 7A, E) and may be due to a limited photoprotective capacity in the mutant. *NPQ* is closely coupled with LHCII, the site of the xanthophyll cycle through which the majority of heat dissipation occurs (Ort, 2001). In the *Y11y11* mutant, chl *b* is greatly reduced and correlates with a 20% reduction in chl per PSI and a 55–65% reduction in chl per PSII, resulting in a severe LHCII truncation that also results in a reduction in the carotenoids necessary for the xanthophyll cycle (Ghirardi and Melis, 1988; Table 1). These deficiencies could have therefore limited the expected increases in *NPQ* levels that should have occurred when light was in excess of carboxylation capacity. Impaired photoprotective mechanisms would be expected to decrease *F*_v_/*F*_m_; however, growth-chamber ambient light levels were less than half of full sunlight and probably prevented severe photoinhibition and a decrease in mutant *F*_v_/*F*_m_ (Table 2).

Growing the two genotypes in the field showed positive effects of reducing chl content on leaf photosynthesis and photoprotection. *ϕPSII* and *q*_P_ were significantly greater in the mutant at moderate light absorption (Fig. 7D, H), indicating slightly greater efficiency in the mutant. In the field mutant, *NPQ* was again lower and *F*_v_′/*F*_m_′ was greater than the WT at mid- to high light levels (Fig. 7B, F), but since these were accompanied by greater *ϕPSII* and *q*_P_, the results imply there was less over-saturation in the light-green leaves at high light as expected. This suggests the more even light distribution in light-green leaves could be increasing photosynthetic capacity and efficiency while decreasing the need for photoprotective mechanisms. These results, in comparison to the results from the chamber experiment, also suggest that a threshold of chl content and LHCII exists for optimal photoprotective mechanisms at high light.

## Conclusions

This study determined that light sheet microscopy is an improved method to examine internal leaf light environments due to enhancing resolution, allowing illumination of either adaxial or abaxial leaf surfaces without additional set-up, and eliminating back-absorption of chl as well as photobleaching of out-of-focus planes. The results revealed a more gradual attenuation of light in the chl mutant as expected. Although measured light attenuation and modeled CO_2_ fixation profiles were more gradual in the mutants and should therefore result in greater photosynthetic capacity and efficiency, pleiotropic effects of the mutation probably prevented any definitive relationships. The severe reduction in mutant chl probably inhibited photosynthetic capacity and efficiency in the chamber experiment, while greater *A*_sat_ and photosynthetic efficiency in the field-grown mutants was confounded with greater *g*_s_. Therefore, further examination of other chl mutants of varying chl reductions is needed to determine if the relationship between light distribution and light-use efficiency still exists in the absence of pleiotropic effects, such as extreme truncation of light-harvesting complexes and increased leaf carbon supply. It is likely that transgenic approaches targeting biosynthetic or regulatory steps in leaf chl accumulation, while avoiding large disturbances in chl *a*/*b* ratios, will be needed to make progress.

## Supplementary Data

Supplementary data are available at *JXB* online.

Fig. S1. Light sheet microscopy set-up.

Fig. S2. Ratio of path length in low-chl plants compared to high-chl plants within species as a function of relative chl content in the low-chl plants.

Fig. S3. Western blots of Rubisco content in WT and *Y11y11* field-grown soybean leaves.

Supplementary Data
