# Supplementary material for: Light sheet microscopy reveals more gradual light attenuation in light-green versus dark-green soybean leaves
Source: J Exp Bot. 2016 Jun 20;67(15):4697–709. doi: 10.1093/jxb/erw246 (PMC4973739; doi:10.1093/jxb/erw246)
Supplement: Supplementary Data [file supp_67_15_4697__index.html]

Light sheet microscopy reveals more gradual light attenuation in light-green versus dark-green soybean leaves — Light sheet microscopy reveals more gradual light attenuation in light-green versus dark-green soybean leaves — Supplementary Data 

# Light sheet microscopy reveals more gradual light attenuation in light-green versus dark-green soybean leaves

## Supplementary Data

Data files

- supplementary\_figures\_S1\_S3.pdf - Supplementary Data
