# Supplementary material for: Light sheet microscopy reveals more gradual light attenuation in light-green versus dark-green soybean leaves
Source: J Exp Bot. 2016 Jun 20;67(15):4697–709. doi: 10.1093/jxb/erw246 (PMC4973739; doi:10.1093/jxb/erw246)
Supplement: Supplementary Data [file supp_erw246_supplementary_figures_S1_S3.pdf]

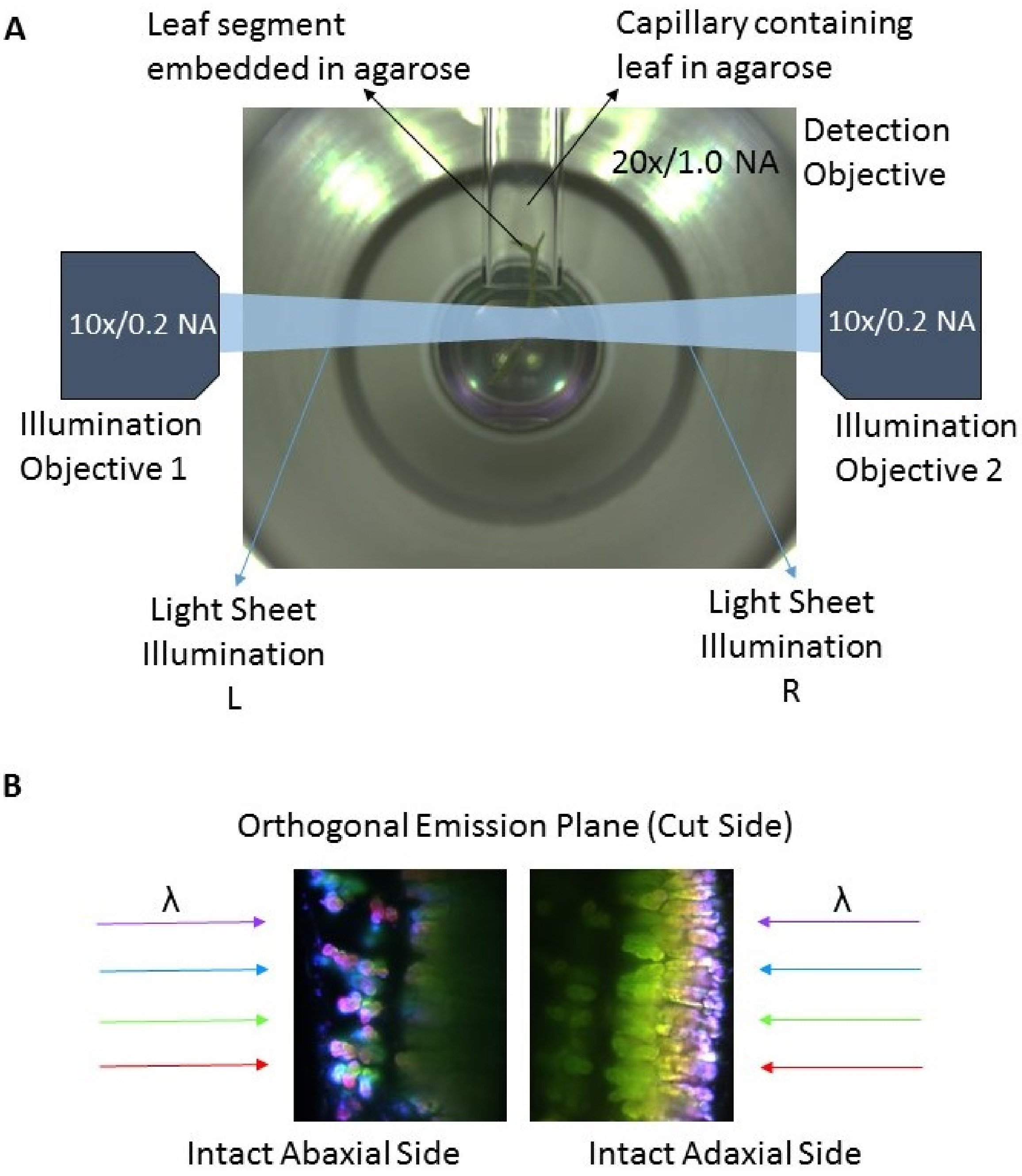

Supplemental Figure 1. Light sheet microscopy setup. A) A leaf segment was inserted into a capillary filled with low-melting and low-gelling agarose. The capillary was rotated until the agarose solidified to keep the sample in the center of the capillar. The capillary was then positioned vertically in a XYZ translational holder and brought into a light sheet chamber filled with ultrapure water. The solidified agarose column was partially ejected from the capillary until the portion of agarose containing the sample was suspended outside of the capillary. The capillary was then rotated so that the adaxial or abaxial sides of the leaf were positioned perpendicular to the illumination objectives and the cut surface was facing the detection objective (positioned orthogonal to the illumination objectives; shown behind the sample). B)

The dual illumination objectives illuminated the tissue with any given wavelength of light (405, 488, 561 and 633 nm) either from left, right, or both sides depending on the experiment. The light sheet illumination angles and the sample positions were calibrated and focused by looking at the chlorophyll fluorescence. The sample then moved across the light sheet to create a Z stack of chlorophyll fluorescence. The illumination was inherently confocal as only the illuminated plane was exposed and emission was detected. The detected chlorophyll fluorescence was captured through the cut surface by the detection objective. Thus, back absorption of light by the chloroplasts was effectively eliminated as the illumination and detection were orthogonal to each other as in the setup described by Vogelmann and Evans (2002).

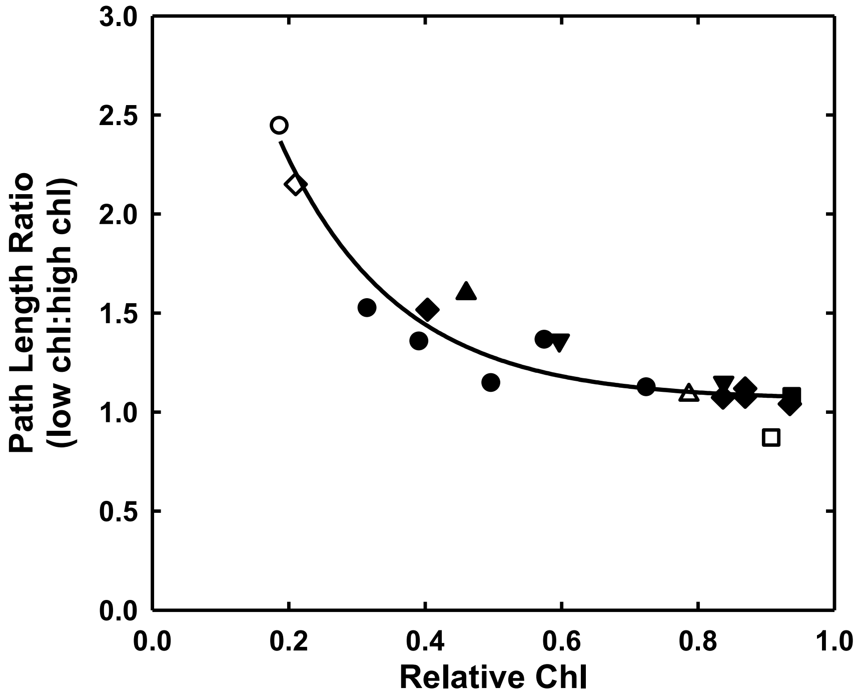

Supplemental Figure 2. Ratio of path length in low chl plants compared to high chl plants within species as a function of relative chl content in the low chl plants. Data are from McClendon and Fukshansky (1990) and the current study. Comparisons were made within the following species: *Catalpa bignonioides* (black circle), *Liriodendron tulipifera* (white circle), *Quercus coccinea* (black down triangle), *Taraxacum* sp. (white triangle), *Nicotiana* sp. (black square), *Nicotiana tabacum* (white square), *Vitis riparia* (black diamond), *Glycine max* (this study: chamber=white diamond, field=black up triangle). The line represents the equation $y=1.06+3.88e^{-5.80x}$ (R^2=0.91).

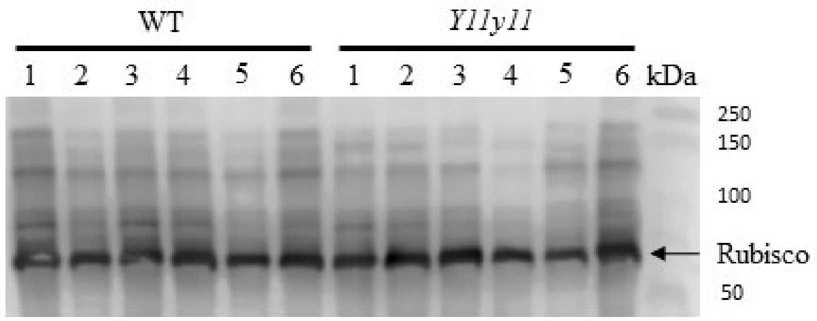

Supplemental Figure 3. Western blots of Rubisco content in WT and *Y11y11* field-grown soybean leaves. A ladder is shown on the far right.
